# Bone Marrow MSC Secretome Increases Equine Articular Chondrocyte Collagen Accumulation and Their Migratory Capacities

**DOI:** 10.3390/ijms23105795

**Published:** 2022-05-21

**Authors:** Romain Contentin, Manon Jammes, Bastien Bourdon, Frédéric Cassé, Arnaud Bianchi, Fabrice Audigié, Thomas Branly, Émilie Velot, Philippe Galéra

**Affiliations:** 1 Normandie University, Unicaen, Biotargen, F-14000 Caen, France; romaincontentin@hotmail.fr (R.C.); manon.jammes@unicaen.fr (M.J.); bastien-bourdon@dielen.fr (B.B.); frederic.casse@unicaen.fr (F.C.); tbranly@gmail.com (T.B.); 2 Molecular Engineering and Articular Physiopathology (IMoPA), French National Center for Scientific Research (CNRS), Université de Lorraine, F-54000 Nancy, France; arnaud.bianchi@univ-lorraine.fr (A.B.); emilie.velot@univ-lorraine.fr (É.V.); 3 Center of Imaging and Research on Locomotor Affections on Equines (CIRALE), Unit Under Contract 957 Equine Biomechanics and Locomotor Disorders (USC 957 BPLC), French National Research Institute for Agriculture Food and Environment (INRAE), École Nationale Vétérinaire d’Alfort, F-94700 Maisons-Alfort, France; fabrice.audigie@vet-alfort.fr

**Keywords:** osteoarthritis, tissue engineering, acellular therapy, horse, mesenchymal stem cells, cell secretome, extracellular vesicles, in vitro repair, cartilage organoids, chondral defects

## Abstract

Equine osteoarthritis (OA) leads to cartilage degradation with impaired animal well-being, premature cessation of sport activity, and financial losses. Mesenchymal stem cell (MSC)-based therapies are promising for cartilage repair, but face limitations inherent to the cell itself. Soluble mediators and extracellular vesicles (EVs) secreted by MSCs are the alternatives to overcome those limitations while preserving MSC restorative properties. The effect of equine bone marrow MSC secretome on equine articular chondrocytes (eACs) was analyzed with indirect co-culture and/or MSC-conditioned media (CM). The expression of healthy cartilage/OA and proliferation markers was evaluated in eACs (monolayers or organoids). In vitro repair experiments with MSC-CM were made to evaluate the proliferation and migration of eACs. The presence of nanosized EVs in MSC-CM was appraised with nanoparticle tracking assay and transmission electron microscopy. Our results demonstrated that the MSC secretome influences eAC phenotype by increasing cartilage functionality markers and cell migration in a greater way than MSCs, which could delay OA final outcomes. This study makes acellular therapy an appealing strategy to improve equine OA treatments. However, the MSC secretome contains a wide variety of soluble mediators and small EVs, such as exosomes, and further investigation must be performed to understand the mechanisms occurring behind these promising effects.

## 1. Introduction

Osteoarthritis (OA) is a worldwide degenerative disease associated with articular pain and decreased articular functional capacities. Cartilage and especially hyaline cartilage markers, such as type II collagen (Col II, are degraded, and the concentration of lubricating molecules in the joint, such as proteoglycan-4 (Prg4), are downregulated [1]. To date, potent curative treatments are not available, but many therapeutic strategies are under investigation. Autologous chondrocyte transplantation (ACT) is one of the most promising therapeutic options [2]. However, ACT requires chondrocytes obtained from biopsies and whose in vitro amplification leads inevitably to cell dedifferentiation. After in vivo implantation, dedifferentiated chondrocytes synthesize a fibrocartilage rich in type I collagen (Col I) fibers which are unable to fully substitute the altered hyaline articular cartilage. This fibrocartilage induction can be considered a transient repair associated with a significant functional recovery of the joint. Nevertheless, fibrocartilage is more quickly degraded upon physiological articular constraints [2,3,4]. In order to improve ACT for cartilage repair strategies, many studies have focused on the chondrogenic potential of stem cells. Among stem cells, the differentiation of adult mesenchymal stem cells (MSCs) into chondrocytes is an attractive approach but has some limitations, notably, their incomplete differentiation status which can generally be improved. Beyond their differentiation capacities, MSCs are able to secrete a large variety of soluble mediators and extracellular vesicles (EVs), known as the cell secretome, which participates in their most relevant physiological functions, including tissue repair and immunomodulation [5,6]. Indeed, several in vivo studies have shown that intra-articular injections of MSCs can promote the repair of chondral defects and/or reduce inflammation [7,8,9,10,11].

Although direct interactions of target cells–MSCs may play a role, the immunomodulatory effect as well as the tissue repair properties of MSCs involve the secretion of soluble mediators [12,13]. Furthermore, a part of the paracrine properties of the MSC secretome also includes the secretion of extracellular vesicles (EVs), notably exosomes [14,15]. Exosomes are small extracellular vesicles (size < 200 nm) expressing cluster of differentiation (CD)9, CD63, CD81, and CD82 tetraspanins and containing endosomal sorting complex required for transport (ESCRT) proteins such as ALIX and tumor susceptibility gene (TSG)101 [16]. Exosomes carry different proteins, miRNAs, and other molecules responsible for cardio-, kidney- and neuroprotective activities, depending on their cell source. They can also exhibit beneficial effects in different diseases such as cerebral ischemia, hepatic fibrosis, and other pulmonary pathologies [15]. Additionally, murine bone marrow MSC (BM-MSC)-derived EVs enhance cartilage molecule expression in vitro and protect cartilage from degradation in an in vivo model of OA [14,17]. On the contrary, human synovial MSC-derived exosomes decreased cartilage extracellular matrix (ECM) marker synthesis but favored chondrocyte proliferation and migration [18].

Co-culture is an in vitro culture method allowing analysis of direct or indirect interaction between two cell types. In the context of ACT, MSCs–chondrocytes co-cultures have been investigated in order to induce MSC chondrogenic differentiation or to improve the phenotype of hyaline articular chondrocytes. For example, seeding simultaneously chondrocytes and MSCs in the same biomaterial leads to a higher production level of cartilage ECM molecules (glycosaminoglycans [GAGs] and collagens) than a biomaterial seeded with a single cell type. In addition, a decreased type X collagen expression, a chondrocyte hypertrophy associated molecule, is observed. The beneficial effect of direct co-culture has not been detected in this study because of the absence of contact between the cells [19]. Another study highlighted the increase in *Col2a1* and SRY [sex-determining region Y]-box 9 (*Sox9*) expression, concomitantly with a decrease in *Col1a1* synthesis, when an indirect co-culture of MSCs and chondrocytes was performed [20]. Besides, it showed that direct co-culture led to the formation of fibrocartilage. The work of Levorson and collaborators also demonstrated that an indirect co-culture of MSCs and chondrocytes generated a higher GAG and collagen production than a direct co-culture [21].

Finally, beyond the phenotypic aspect of chondrocytes, the use of MSCs in co-culture may be relevant to improve chondrocyte proliferation and survival. Indeed, the MSC secretome decreased the expression of pro-apoptotic molecules by cardiomyocytes in a model of myocardial infarction in rats [22]. Additionally, a co-culture of human MSCs derived from adipose tissue and chondrocytes reduced chondrocyte apoptosis [23].

The aim of this study is to assess new potential therapeutic strategies to limit the impact of OA in horses, as it remains a challenge for the equine industry [24]. As explained above, co-culture seems to be an appropriate method to improve chondrocyte phenotype, survival, and proliferation in the context of ACT. Nevertheless, further studies are required to identify whether indirect co-culture (MSCs–chondrocytes) improves the chondrocyte phenotype in an equine model. The effectiveness of indirect co-culture could improve chondrocyte phenotype thanks to an incubation with MSC-conditioned media (MSC-CM) or MSC-derived EVs/exosomes.

In this study, we first assessed the indirect effect of MSC co-culture on equine articular chondrocytes (eACs) seeded in a monolayer (a two-dimensional [2D] system) or in a 3D (three-dimensional) scaffold, as previously described [25,26,27]. Then, we used MSC-CM in order to enhance the quality of cartilage organoids, and we also investigated its effect on chondrocyte migration. Ultimately, we showed that MSC-CM contains numerous small particles of nanometric size and a morphology compatible with small EVs, such as exosomes.

## 2. Results

### 2.1. MSCs Increase eAC mRNA Levels of Hyaline Cartilage and Fibrocartilage Markers Collagens, Prg4 and Proliferation Associated Molecules

**Figure 1:**
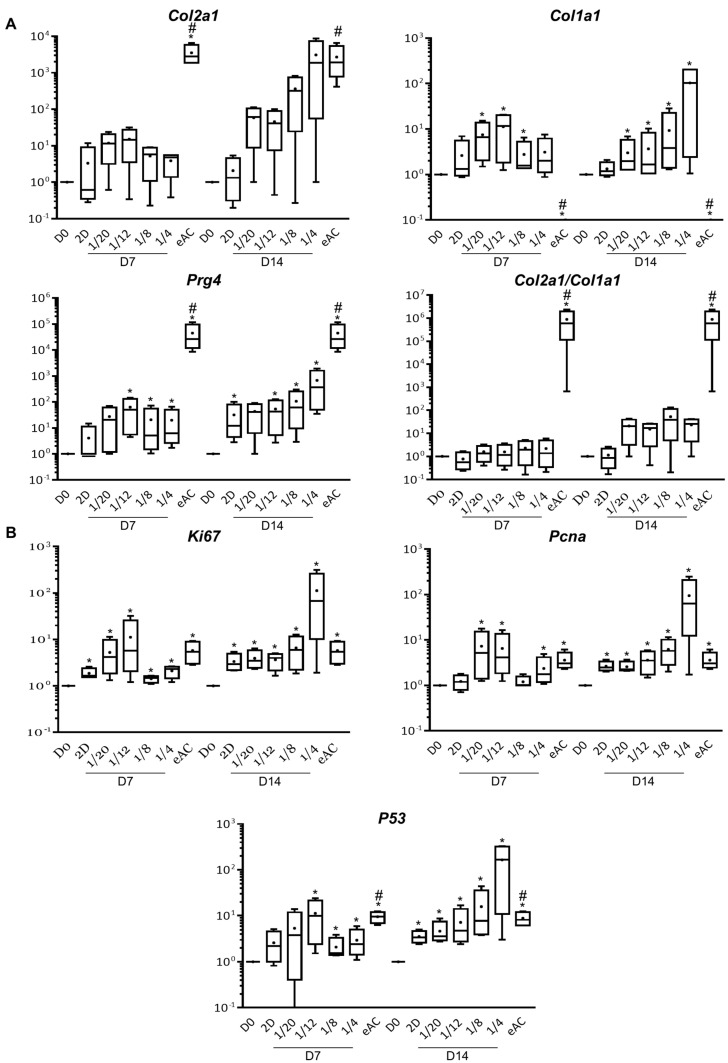
Analysis of eAC mRNA levels of hyaline cartilage and fibrocartilage markers, and proliferation-associated molecules when eACs were seeded in monolayer and co-cultured with MSCs at several ratios for 7 or 14 days. At P3, eACs were seeded in monolayer. Then, MSCs were co-cultured in an upper chamber for 7 or 14 days to obtain MSCs/eACs ratios from 1/20 to 1/4. Total mRNA was extracted and RT-qPCR was performed to assess the mRNA levels of hyaline cartilage and fibrocartilage markers (types II and I collagens, and *Prg4*) (**A**) and proliferation-associated molecules (*Ki67*, *Pcna*, and *P53*) (**B**). The D0 condition corresponds to eACs cultured in monolayer until P3. The eAC condition corresponds to eACs at P0. mRNA levels were estimated by RT-qPCR after normalization relative to the *β*-*actin* reference gene. The results (*n* = 4) are represented as box plots (median, quartiles, extreme values) and the statistical significance of the values was tested using the Mann–Whitney test with the D0 condition (* *p* < 0.05) and the 2D condition (# *p* < 0.05) as references. 2D-for cells cultured in monolayer; *Col1a1*-gene encoding the alpha1(I) mRNA of type I collagen; *Col2a1*-gene encoding the alpha1(II) mRNA of type II collagen; D0-for untreated eACs; D7-time point corresponding to the cells cultured for 7 days; D14-time point corresponding to the cells cultured for 14 days; eACs-equine articular chondrocytes; *Ki67*-gene encoding the *Ki67* mRNA, MSCs-mesenchymal stem cells; P0-primary culture; P3-passage 3; *P53*-gene encoding the *P53* mRNA; *Pcna*-gene encoding proliferating cell nuclear antigen mRNA; *Prg4*-gene encoding proteoglycan 4; RT-qPCR-reverse transcription-quantitative polymerase chain reaction.

We first assessed the effect of indirect co-culture of MSCs-eACs with chondrocytes at passage 3 (P3) seeded in monolayer and cultured for 7 (D7) or 14 days (D14) (Figure 1). When eACs were cultured alone with the 2D amplification medium (referred to as “2D”), the mRNA levels of *Col1a1*, *Col2a1*, and *Prg4* tended to increase without reaching the statistical significance (except for *Prg4* at D14), compared to the untreated condition (D0) (Figure 1A). On the contrary, when eACs were co-cultured with MSCs, the mRNA levels of *Col1a1* and *Prg4* were significantly increased compared to D0. After 7 days of culture, the mRNA levels of *Col2a1* tended to increase and, after 14 days of culture (for the 1/4 ratio), reached similar levels to the undifferentiated eAC control (at P0). However, mRNA levels of *Col1a1*, *Col2a1*, and *Prg4* were not statistically different between eACs co-cultured with MSCs and eACs cultured alone (2D condition). We observed only trends. Indeed, at D7, the 1/20 and 1/12 ratios tended to lead to higher eAC mRNA levels of type I and type II collagens than eACs cultured alone (2D condition). All the ratios studied at D7 tended to increase the mRNA levels of *Prg4*, compared to the 2D condition. At D14, mRNA levels of both type I and type II collagens tended to increase whatever the MSCs/eACs ratio compared to the 2D condition, whereas mRNA levels of *Prg4* were increased only for the 1/8 and 1/4 ratios. Thus, the functional *Col2a1*/*Col1a1* ratio, whose elevated value is correlated with chondrocytes having a hyaline articular phenotype, was increased when eACs were co-cultured with MSCs only after 14 days of culture, but here again, the differences observed were not statistically significant (Figure 1A).

The mRNA levels of proliferation-associated molecules *Ki67* and proliferating cell nuclear antigen (*Pcna*) increased statistically significantly compared to D0 and tended to increase when eACs were co-cultured with MSCs for 7 days (only for the 1/20 and 1/12 ratios) and 14 days (only for the 1/8 and 1/4 ratios), compared to eACs cultured alone (2D condition) (Figure 1B). Furthermore, *P53* mRNA levels followed the same trend.

**Figure 2:**
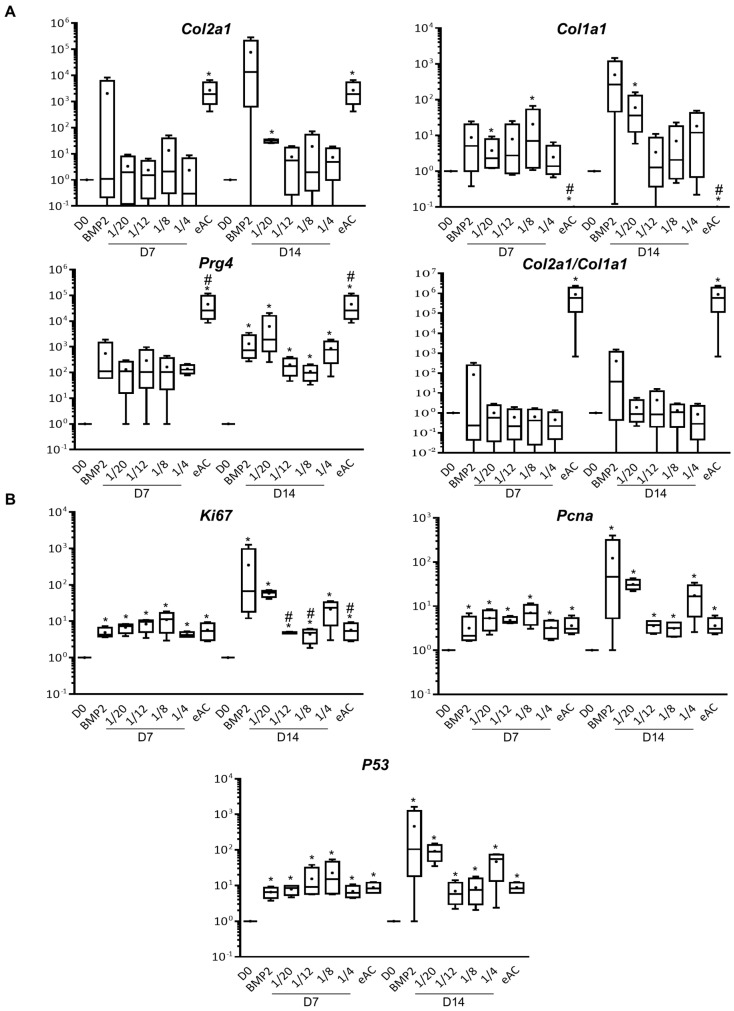
Analysis of eAC mRNA expression of hyaline cartilage and fibrocartilage markers, and proliferation-associated molecules when eACs were seeded in a 3D scaffold and co-cultured with MSCs at different ratios for 7 or 14 days. eACs were seeded in 3D with type I/III collagen sponges at 800,000 cells/sponge. Then, MSCs were seeded in an upper chamber for 7 or 14 days to obtain MSCs/eACs ratios from 1/20 to 1/4. Total mRNA was extracted and RT-qPCR was performed to assess the mRNA levels of hyaline cartilage and fibrocartilage markers (types II and I collagens, and *Prg4*) (**A**) and proliferation-associated molecules (*Ki67*, *Pcna*, and *P53*) (**B**). The D0 condition corresponds to eACs cultured in monolayer until P3. The eAC condition corresponds to eACs at P0. BMP-2 condition corresponds to eACs cultured in 3D in the presence of BMP-2 (50 ng/mL). mRNA levels were estimated by RT-qPCR after normalization relative to the *β*-*actin* reference gene. The results (*n* = 4) are represented as box plots (median, quartiles, extreme values) and the statistical significance of the values was tested using the Mann–Whitney test with the D0 condition (* *p* < 0.05) and the BMP-2 condition (# *p* < 0.05) as references. Note: 3D-for cells cultured in type I/III collagen sponges; BMP-2-bone morphogenetic protein 2; *Col1a1*-gene encoding the alpha1(I) mRNA of type I collagen; *Col2a1*-gene encoding the alpha1(II) mRNA of type II collagen; D0-for untreated eACs; D7-time point corresponding to the cells cultured for 7 days; D14-time point corresponding to the cells cultured for 14 days; eACs-equine articular chondrocytes; *Ki67*-gene encoding the *Ki67* mRNA, MSCs-mesenchymal stem cells; P0-primary culture; P3-passage 3; *P53*-gene encoding the *P53* mRNA; *Pcna*-gene encoding proliferating cell nuclear antigen mRNA; *Prg4*-gene encoding proteoglycan 4; RT-qPCR-reverse transcription-quantitative polymerase chain reaction.

When eACs were cultured as cartilage-like organoids, co-culture with MSCs induced a statistically significant increase in mRNA levels of type I collagen for the 1/20 ratio after 7 and 14 days of culture as well as for the 1/8 ratio at D7 (Figure 2A). Regardless of the culture time, the co-culture eACs-MSCs at the other ratios tended to increase the eAC mRNA levels of *Col1a1* compared to D0, to reach similar levels (D7) than those observed when eACs were cultured in presence of a potent chondrogenic activator of collagen expression as previously described [28], bone morphogenetic protein-2 (BMP-2), or lower levels at D14. The mRNA levels of type II collagen also tended to increase in the presence of MSCs at D7 and D14, compared to D0 but remained below the controls cultured in the presence of BMP-2 and eAC controls (P0). As a result, when eACs were co-cultured with MSCs, the *Col2a1/Col1a1* ratio was not modulated compared to D0 and was lower than eAC controls. On the contrary, when eACs were grown in the presence of MSCs, the mRNA levels of *Prg4* were closely similar to controls cultured in the presence of BMP-2 and were significantly increased at D14, compared to D0. eAC mRNA levels of *Ki67* and *Pcna* were increased, whatever the MSCs/eACs ratio, after 7 days and 14 days of culture, compared to D0 (Figure 2B). Nevertheless, after 14 days of culture, these mRNA levels remained lower than those of eACs cultured in presence of BMP-2. mRNA levels of *P53* followed the same trend. Altogether, these results demonstrate that the MSC secretome modulates eAC mRNA levels of *Col2a1*, *Col1a1*, *Prg4*, *Ki67*, *Pcna*, and *P53*.

### 2.2. MSC-CM Are Not Cytotoxic and do Not Affect eAC Proliferation

**Figure 3:**
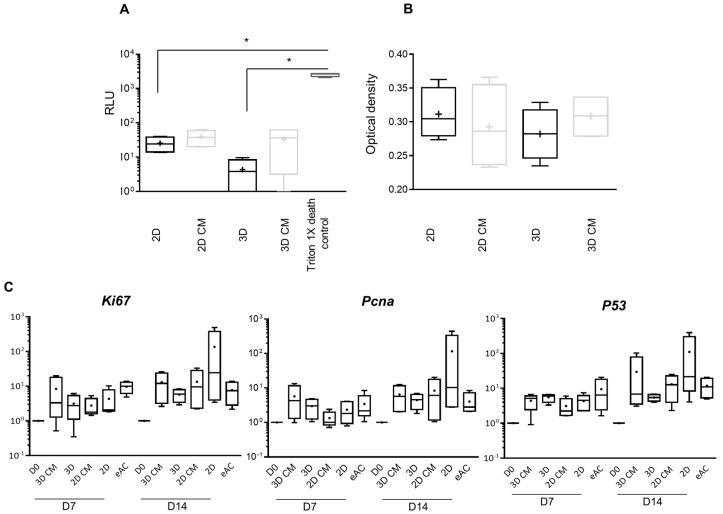
MSC-CM is not cytotoxic and does not affect eAC proliferation. eACs were seeded in 3D with type I/III collagen sponges or in 2D in monolayer until their confluency reached 80% then treated for 72 h to evaluate MSC-CM potential cytotoxicity with ToxiLight^TM^ assay (**A**) and measure cell metabolic activity with XTT assay (**B**). For gene expression analysis, eACs were seeded in 3D with type I/III collagen sponges or in 2D in monolayer then treated 24 h later for 7 or 14 days (**C**). Total mRNA was extracted and RT-qPCR was performed to assess the mRNA levels of proliferation-associated molecules (*Ki67*, *Pcna*, and *P53*). The D0 condition corresponds to eACs cultured in monolayer until P3. The eAC condition corresponds to eACs at P0. mRNA levels were estimated by RT-qPCR after normalization relative to the *β-actin* reference gene. The results (*n* = 4) are represented as box plots (median, quartiles, extreme values) and the statistical significance of the values between MSC-CM and respective control media was tested using the Mann–Whitney test (* *p* < 0.05). Note: 2D-for medium used for chondrocyte amplification; 2D CM-for 2D-conditioned medium incubated with MSCs for 24 h and filtered; 3D-for medium used for chondrocyte redifferentiation; 3D CM-for 3D-conditioned medium incubated with MSCs for 24 h and filtered. D0-corresponds to untreated eACs cultured in monolayer until P3. D7-time point corresponding to the cells cultured for 7 days; D14-time point corresponding to the cells cultured for 14 days; eACs-equine articular chondrocytes; *Ki67*-gene encoding the *Ki67* mRNA, MSCs-mesenchymal stem cells; P0-primary culture; P3-passage 3; *P53*-gene encoding the *P53* mRNA; *Pcna*-gene encoding proliferating cell nuclear antigen mRNA; RLU-relative luciferase unit; RT-qPCR-reverse transcription-quantitative polymerase chain reaction; Triton 1X death control-eACs cultured for 72 h in 2D medium, then Triton 1X is added at 72 h to induce cell death.

We have assumed that preparation of MSC-CM, in order to incubate them with chondrocytes cultured in monolayer (2D CM) or in 3D (3D CM), could have similar effects to what we observed during indirect co-culture of MSCs/eACs. Thus, we first assessed the effect of MSC-CM on the cytotoxicity and metabolic activity of eACs (Figure 3). Two-dimensional and 3D CM were not cytotoxic for eACs (Figure 3A) as adenylate kinase (AK) activity levels were not different from 2D and 2D classical control media (not conditioned), and did not enhance the metabolic activity of eACs (Figure 3B). mRNA levels of *Ki67* and *Pcna* tended to slightly increase when eACs were cultured with 3D CM and tended to decrease when eACs were cultured in presence of 2D CM compared to respective control media (Figure 3C). mRNA levels of *P53* were not modulated by MSC-CM. Altogether, these results showed that the MSC-CM does not significantly affect eAC proliferation.

### 2.3. MSC-CM Favor Collagen Synthesis

**Figure 4:**
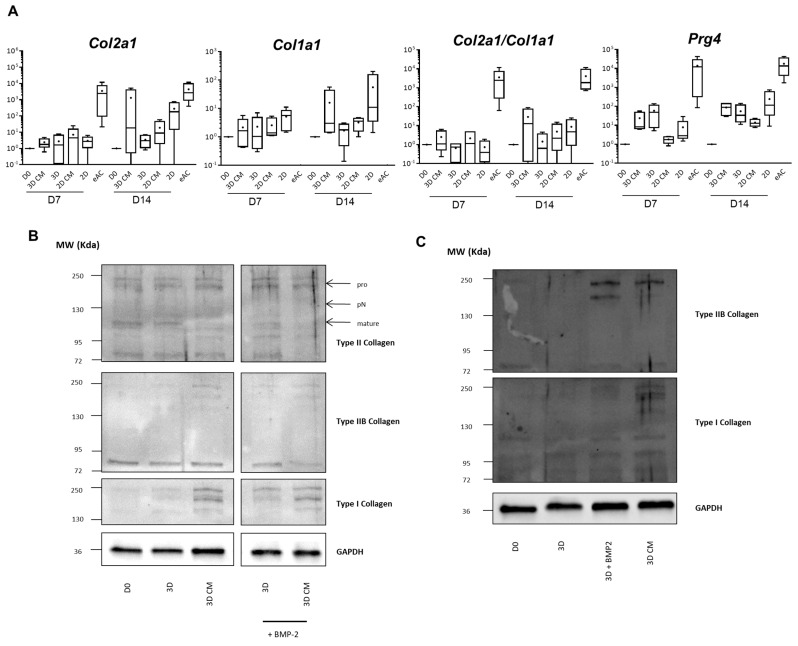
Effect of MSC-CM on the steady-state mRNA levels of some cartilage ECM molecules in eACs cultured in a 3D scaffold or in monolayer. eACs were seeded in 3D with type I/III collagen sponges or in 2D in monolayer then treated 24 h later for 7 or 14 days. The D0 condition corresponds to eACs cultured in monolayer until P3. Total mRNA was extracted and RT-qPCR was performed to assess the mRNA levels of hyaline cartilage and fibrocartilage markers (*Col2a1*, *Col1a1*, and *Prg4*) (**A**). The D0 condition corresponds to eACs cultured in monolayer until P3. The eAC condition corresponds to eACs at P0. mRNA levels were estimated by RT-qPCR after normalization relative to the *β*-*actin* reference gene. The results (*n* = 4) are represented as box plots (median, quartiles, extreme values) and the statistical significance of the values between MSC-CM and respective control media were tested using the Mann–Whitney test. Proteins were extracted and Western blots were performed to evaluate the protein presence of type I, type II and IIB collagens, as shown in the representative pictures (**B**,**C**). Only the expression of proteins at D14 are shown. The numbers on the left of the blots represent the molecular weight markers expressed in kDa. Note: 2D-for medium used for chondrocyte amplification; 2D CM-for 2D-conditioned medium incubated with MSCs for 24 h and filtered; 3D-for medium used for chondrocyte redifferentiation; 3D CM-for 3D-conditioned medium incubated with MSCs for 24 h and filtered. BMP-2-bone morphogenetic protein 2; *Col1a1*-gene encoding the alpha1(I) mRNA of type I collagen; *Col2a1*-gene encoding the alpha1(II) mRNA of type II collagen; D0-time point corresponds to untreated eACs cultured in monolayer until P3. D7-time point corresponding to the cells cultured for 7 days; D14-time point corresponding to the cells cultured for 14 days; eACs-equine articular chondrocytes; GAPDH-glyceraldehyde-3-phosphate dehydrogenase; MSCs-mesenchymal stem cells; P0-primary culture; P3-passage 3; *Prg4*-gene encoding proteoglycan 4; RT-qPCR-reverse transcription-quantitative polymerase chain reaction.

eACs were seeded in a 3D collagen scaffold and cultured in the presence of 3D CM or they were cultured as monolayers in the presence of 2D CM (Figure 4A). After 7 days of culture, the mRNA levels of *Col2a1*, *Col1a1*, and *Prg4* were similar between eACs cultured with MSC-CM and eACs cultured with control media. Nevertheless, the *Col2a1/Col1a1* ratio tended to be slightly increased in presence of MSC-CM. After 14 days of culture, 3D CM seemed to increase the mRNA levels of both *Col2a1* and *Col1a1*, and the *Col2a1/Col1a1* ratio was also enhanced. When eACs were cultured in a monolayer with 2D CM, mRNA levels of type I and type II collagens tended to decrease compared to eACs grown in presence of 2D medium, but the *Col2a1/Col1a1* ratio remained unchanged nonetheless. mRNA levels of *Prg4* remained unchanged with 3D medium but decreased in the presence of the 2D CM. However, only trends and no statistically significant differences were observed. Then, we evaluated the collagen protein amounts in the organoids generated by eACs cultured in a 3D collagen scaffold for 7 (Figures S1–S3) or 14 days with 3D medium or 3D CM (Figure 4B,C, Figures S1–S6). The accumulation of the collagens was very low in the control condition (3D) after 7 or 14 days of culture. The addition of BMP-2 led to an increase in total type II and type IIB collagens, which are hyaline articular cartilage typical collagens, and fibrocartilage associated type I collagen. Regardless of the culture time, 3D CM led to an increased accumulation of type I, total type II, and type IIB collagens even though the IIB isoform remained weakly expressed. Altogether, these results showed that MSC-CM enhances type II and type I collagen syntheses of eACs cultured as cartilage-like organoids.

### 2.4. eACs Cultured in the Presence of MSC-CM have Increased Migratory Capacities

**Figure 5:**
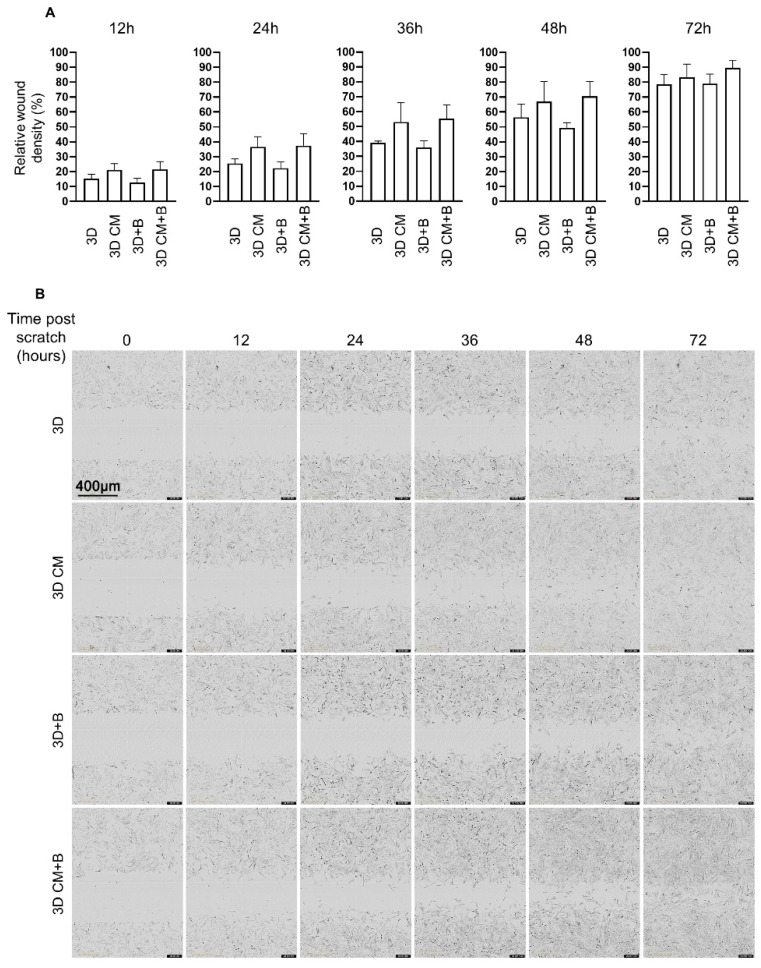
eACs cultured in presence of MSC-CM have increased migratory capacities. eACs were seeded in 2D in monolayer and a scratch was made after 30 h. Then, the cells were treated until 72 h. The results (*n* = 4) are represented as histograms (mean + standard deviation) and the statistical significance of the values between MSC-CM and respective control media were tested using the Mann–Whitney test (no statistical significance was observed) (**A**). Photographs are representative wells of the experiments (the scale bar = 400 µm presented in the upper left photograph is the same for all the other photographs) (**B**). Note: 3D-for medium used for chondrocyte redifferentiation; 3D CM-for 3D-conditioned medium incubated with MSCs for 24 h; B-for BMP-2 supplementation; BMP-2-bone morphogenetic protein-2; eACs-equine articular chondrocytes; MSC-mesenchymal stem cell; MSC-CM-mesenchymal stem cell-conditioned media.

eACs were cultured as monolayers and when confluency was reached, an area of each well was scratched. The time and the way that cells were able to refill the wound was analyzed to assess the effect of 3D CM. Whatever the timepoint post-scratch, the proportion of eACs in the initial scratch area was increased when the cells were cultured with MSC-CM, in the presence of BMP-2 or not (a potent chondrogenic activator as previously described [28], used as a control) compared to eACs grown in control media (Figure 5A). These data indicate that BMP-2 did not induce any additive effect with MSC-CM. Additionally, 24 h post-scratch, eACs at the edges of the scratch exhibit a spindle-shaped morphology only in the presence of MSC-CM (Figure 5B). The wound closure was advanced when eACs were cultured with MSC-CM compared to eACs cultured in control media. Indeed, in the presence of MSC-CM, most of the wound area has been colonized 48 h post-scratch with the spindle-shaped eACs, whereas 72 h is needed for the eACs cultured with control media. These results prove that 3D CM from MSCs is able to enhance the migratory capacities of eACs with a noticeable modification of their morphology.

### 2.5. MSC-CM Contain Small Particles

**Figure 6:**
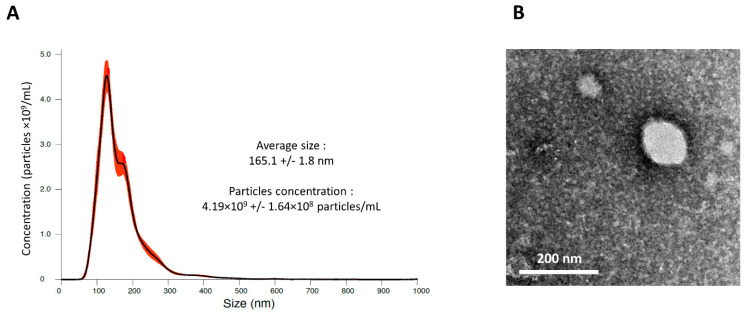
MSC-CM contains undamaged small EVs from BM-MSCs. Conditioned media of equine BM-MSCs were harvested after 24 h of exposure. Then, samples were diluted and prepared to be processed for physical and morphological evaluation of the particles contained in MSC-CM. MSC-CM nanoparticle size distribution and concentration were determined by NTA (**A**). MSC-CM particle morphology was observed by TEM to confirm their EV nature and assess EV integrity (**B**). BM-MSCs-bone marrow-mesenchymal stem cells; MSC-CM-mesenchymal stem cell-conditioned media; EVs-extracellular vesicles; NTA-nanoparticle tracking analysis; TEM-transmission electron microscopy.

The MSC secretome is composed of a wide variety of molecules. Nevertheless, since it has already been demonstrated that EVs derived from MSCs were able to enhance chondrocyte proliferation, delay experimentally induced OA, and even repair cartilage [6], we hypothesized that equine MSC-derived EVs could participate in the effect we observed with eACs. The size and concentration of nanoparticles included in MSC-CM were assessed by nanoparticle tracking analysis (NTA). We identified that equine MSC-CM contains nanoparticles with an average size below 200 nm and with a concentration close to 4 billion particles/mL (Figure 6A). Transmission electron microscopy (TEM) micrographs confirmed the nanoscale size of these particles and showed that they had a vesicular morphology in line with EVs with a well-defined dark membrane boundary and relatively dense content (Figure 6B). However, further investigations are still needed to fully characterize MSC-CM-derived nanoscale EVs.

## 3. Discussion

The goal of this study was to assess the effect of the MSC secretome on eAC phenotype. We initially thought, like others, that the MSC secretome would be of interest to improve eAC phenotype in the context of equine OA. New strategies to treat equine OA are still needed since a high proportion of athlete horses undergo articular disorders which can lead to an early career cessation, tremendous financial loss, and impaired horse well-being [24,29,30]. Furthermore, the joint anatomy, physiopathology of OA, and composition of articular cartilage and thickness are closely similar between horses and humans. Thus, advances in horse OA treatment could be directly transposable to humans according to the “One Health” principle [31].

We found that the MSC secretome led to an increase in the eAC collagen protein amounts, mRNA levels of *Prg4*, and enhanced proliferation and migratory capacities of eACs. Nevertheless, we have noticed that the phenotype of eACs cultured in the presence of MSC-CM and the phenotype of eACs co-cultured with MSCs were slightly different. Notably the mRNA levels of *Prg4* which was increased when eACs were co-cultured with MSCs, whereas CM did not induce any modification of *Prg4* steady-state levels. *Prg4* is highly expressed by synoviocytes and superficial chondrocytes [32]. Besides its roles in lubrication, Prg4 is able to bind and regulate the toll-like receptors, and hence inflammatory downstream pathways [33]. Interestingly, cartilage *Prg4* expression decreased in an equine OA model [34]. Additionally, the proliferation-associated molecules tended to increase only when eACs were co-cultured in the presence of MSCs. This effect was not retrieved when eACs were cultured with MSC-CM. On the contrary, the upregulation of eAC types II and I collagen amounts was systematically observed, either when eACs were co-cultured with MSCs or incubated with MSC-CM. Furthermore, a previous study has already shown that CM from adult articular chondrocytes combined with a biomaterial scaffold is able to induce chondrogenic differentiation of human BM-MSCs and Wharton’s jelly-MSCs, leading to ECM synthesis [35]. Altogether, these results suggest that the chondrocyte secretome affects MSCs and their own secretome, which induces in return beneficial effects on eAC phenotype. Further investigations are needed to identify the molecules involved in this mechanism, since the chondrocyte secretome contains a wide variety of molecules and EVs, such as exosomes [36].

When we prepared CM, we collected molecules secreted by MSCs for a limited period of time (24 h) and then incubated eACs with the same MSC-CM for at least 3 days. On the contrary, co-cultures allowed the eACs to be under a continuous supply of MSC secretome throughout the culture time. Thus, it is possible that eACs co-cultured with MSCs were grown with a higher amount of MSC secretome active molecules than eACs cultured with MSC-CM, which could account for the data differences we observed between these two culture approaches.

We also assessed the toxicity of MSC-CM on eACs, because it has been shown that MSC-CM could be toxic, notably for an organotypic culture of rat hippocampus [37]. Here, we unambiguously demonstrated that MSC-CM was not toxic for eACs culture and can be used for further experiments.

OA is an evolutive disease characterized by sequential events which ineluctably ends up in total cartilage destruction. Indeed, following the events that trigger OA, chondrocytes increase their proliferation and synthesize an atypical ECM, notably composed of type I collagen [38]. The neosynthesized fibrocartilage is more quickly degraded leading to increased inflammation, chondrocyte apoptosis, and bone end exposure. Nevertheless, the fact that MSC-CM significantly increased the synthesis of type I collagen by eACs in our study is promising. Indeed, in the context of OA, enhanced ECM synthesis would delay the painful bone frictions that occur at the latest stage of the disease. Furthermore, renowned equine clinicians agree that this enhancement in ECM synthesis, including type I collagen, is favorable as it allows functional recovery of the joint in horses, particularly in sport horses whose career is short, and this reduces the latency for a faster return to competition. For all horses, sporting or not, this functional recovery of the joint, even if it can be transient, has necessarily beneficial effects if we take into consideration the life span, which is much shorter in horses than in humans. Although similar effects with MSC-CM on the biosynthesis of type II collagen may appear to be of lesser magnitude, this is most certainly due to the fact that the anti-type I collagen antibody is of the highest possible quality (it has a very high titer), which is not at all the case for the anti-collagen II antibody. Nevertheless, these effects will have to be confirmed by carrying out, for example, enzyme-linked immunoassay (ELISA) assays on cartilage organoid cultures, considering the fact that type I collagen is a more soluble isotype in cell culture (present in the culture medium), whereas type II collagen is an integral part of the insoluble fraction that associates mainly with the ECM set up within cartilage organoid.

The OA pathogenesis is related to whole joint inflammation and a decrease in the lubricating molecules concentration in the joint [1]. In this context enhancing chondrocyte proliferation, collagen syntheses, and maintaining lubricating molecule expression, such as Prg4, under MSC-CM treatment are of interest to delay the final outcomes of OA.

When we performed the wound healing assay, we showed that eACs cultured with MSC-CM exhibited a spindle shape and fulfilled more rapidly in the wound area whereas the MSC-CM had no effect on eAC proliferation (as shown with the metabolic activity assay and the study of proliferation-associated molecule mRNA levels). Altogether, this shows that MSC-CM favors in vitro eAC migration. When a chondral defect occurs in vivo, cell progenitors and chondrocytes surrounding the defected area have difficulties migrating and fulfilling the chondral loss. Thus, in vivo, MSC-CM could favor chondrocytes and progenitors’ migration to facilitate the repair of the defect. On the contrary, this eAC shape modification can indicate a split from the initial cell type leading to a difference in eAC properties and the nature of ECM synthesis. In vitro 2D culture-associated dedifferentiated phenotype is characterized by a fibroblastic-like shape, as was observed in these experiments. It has been shown that eACs can also degenerate in vivo into different phenotypes including dedifferentiated-like ones [39], hence this phenomenon could also happen during in vivo treatment.

In order to optimize/increase the beneficial effects of MSC-CM on eACs phenotype, several strategies have been considered, such as the priming of MSCs. Indeed, pre-conditioning MSCs, prior to their in vivo injection, increases their survival and therapeutic efficacy [40]. MSCs can be primed with, amongst others, cytokines, chemical agents, hypoxia, and culture conditions [41]. The priming of MSCs with pro-inflammatory cytokines is taking a growing interest due to the efficiency and ease of this method. Interferon-γ (IFN-γ) enhanced the immunosuppressive properties of MSCs, notably by upregulating immunosuppressive factors and chemokine ligands. Tumor necrosis factor-alpha (TNF-α) priming led to an upregulation of immunoregulatory factors. The priming of MSCs with several inflammatory cytokines may have additional effects, as shown with the combination of IFN-γ and TNF-α in a recent study [42]. Human MSCs stimulated with interleukin (IL)-1β, IL-6, and IL-23 produced higher levels of transforming growth factor-beta (TGF-β) than the unstimulated MSCs [43]. Growth factors are also able to influence the MSC secretome. Thus, the MSC secretome must be optimized in order to enhance the eAC phenotype.

Oxygen availability during in vitro expansion is classically higher compared to bone marrow. Under hypoxic conditions, MSCs secrete a high content of soluble bioactive factors and have a strong regenerative potential. Thus, an incubation of MSCs in hypoxia to mimic in vivo MSC niche conditions may enhance their proliferation, and their immunomodulatory and regenerative potentials [41].

MSCs are able to secrete a wide variety of soluble mediators and EVs. It is likely that the combination of several molecules/EVs of the equine MSC secretome led to the beneficial effects on the eAC phenotype we observed. We demonstrated that MSC-CM contains nanoscale particles which could be exosomes since we detected EVs characterized by the presence of a black-colored plasma membrane with an irregular appearance of the contents, a characteristic of exosomes. Similarly, the nanoparticles detected are closer in size to those of exosomes. Thus, exosomes could have a preponderant role in the effect of the MSC-CM we observed. A future study of the MSC-CM vesiculome, comprising EVs secreted by MSCs, could confirm our hypothesis. Indeed, several works in small mammal models have demonstrated that EVs derived from multipotent stem cells were able to enhance chondrocyte proliferation, delay experimentally induced OA, and even restore cartilage [6]. Additionally, EVs can have an equivalent or even a superior effect than the cell type they derive.

Current ACT procedures are limited by chondrocyte dedifferentiation and the perfectible redifferentiation. Unfortunately, our result suggests that MSC-CM, as we used it in this study, could not increase eAC proliferation during the amplification step of ACT. Further investigation is required to fully conclude the interest of MSC-CM (as we prepared them in our study) to improve the differentiation state of eACs. Indeed, an incubation of eACs with BMP-2 (which recapitulates at least in part a pro-chondrogenic environment [28]) and MSC-CM led systematically to an increase in type I collagen biosynthesis, but also in type II and IIB collagen production, compared to eACs cultured with BMP-2 alone. Nevertheless, the *Col2a1/Col1a1* ratio was not increased when eACs were co-cultured with MSCs. Thus, to consider the utility of MSC-CM during the in vitro part of the ACT procedure, an optimization of the MSC-CM use would be required. In fact, the 2D amplification of this cell type may not be appropriate, and getting closer to the in vivo conditions with 3D culture, hypoxia, or mechanical stimulation could improve MSC-CM regenerative effects on eACs [44]. For instance, it has been demonstrated that hypoxic culture conditions can stimulate hepatocyte growth factor (HGF) and vascular endothelial growth factor (VEGF) secretion by human MSCs, improving their neuroregenerative properties in brain injury therapy [45]. This study showed the importance of culture conditions for the MSC secretome quality and by extension for their efficiency in regenerative treatments.

To date, many therapeutic strategies have been investigated to avoid/delay OA outcomes. Indeed, intra-articular injection of hyaluronan, corticosteroids, polysulfated glycosaminoglycan, and autologous-conditioned serum may help to delay cartilage degradation and reduce the symptoms of OA [46]. Besides intra-articular injection, some medications can be topically administered and nutraceuticals may also be of interest in order to slow down OA progression [47]. Whatever the pathophysiological mechanism disrupted by the current treatments, the long-term OA outcomes are ineluctable. In this context, the MSC features offer many opportunities to improve current therapeutic strategies for the treatment of chondral lesions. Indeed, beyond the chondrogenic differentiation potential of MSCs, their secretome could be a therapeutic alternative. MSC injection in the equine model led to a reduction in the progression of OA [11]. Nevertheless, concomitantly with allogeneic MSCs or even autologous injections, and despite their immunomodulatory properties, MSCs can stimulate innate immune responses which can result in deleterious inflammatory reactions [10,48,49]. Even if it has not been noticed yet, malignant transformation is also a risk for all cell therapies. The fate of the injected MSCs is also of concern as they survive for a short period within the joint. Our study has demonstrated that the MSC secretome makes the acellular therapy possible to overcome the MSC limitations while keeping their therapeutic potential in the context of equine OA.

## 4. Materials and Methods

### 4.1. Cell Isolation and Culture

Bone marrow-derived MSCs were isolated and characterized from horses with ages ranging from 3 to 4 years old, as previously described [50,51,52,53]. Briefly, we performed gradient centrifugation with Ficoll-Paque PREMIUM (GE healthcare Bio-sciences; Chicago, IL, USA), then collected and seeded the interphase in a plastic flask in the presence of an isolation medium (Low Glucose-Dulbecco’s Modified Eagle’s Medium (LG-DMEM; Invitrogen; Carlsbad, CA, USA) containing 30% fetal calf serum (FCS, Invitrogen Life Technologies; Carlsbad, CA, USA), 10^−7^ M dexamethasone (Sigma-Aldrich; Saint Louis, MO, USA). Within a few days at 37 °C and 5% CO_2_, MSCs formed colonies. Colonies were trypsinized (Invitrogen; Carlsbad, CA, USA), seeded (5000 cells/cm^2^, P1), and amplified in LG-DMEM medium containing 20% FCS (MSC amplification medium). Antimicrobial agents were added to all the culture media (100 IU/mL of penicillin, 10 mg/mL of streptomycin, and 0.025 mg/mL of amphotericin B [PSA], Eurobio Scientific; Les Ulis, France). Proliferative capacities, multipotency, and the presence/absence of surface markers (CD29, CD44, CD45, CD73, CD90, CD105, and type II major histocompatibility complex [MHC]) on the isolated cells were analyzed for the characterization of MSCs.

eACs were isolated from the cartilage of carpal and femoral condyles from horses aged from 4 to 10 years old. The visually healthy parts were dissected to obtain chips a few millimeters thick. The chondrocytes were then isolated by successive digestions, at 37 °C, for 45 min with 2 mg/mL of *Streptomyces griseus* protease (Sigma-Aldrich; Saint Louis, MO, USA) then 18 h in the presence of 2 mg/mL of *Clostridium histolyticum* type I collagenase (Invitrogen Life Technologies; Carlsbad, CA, USA). Both enzymes were diluted in DMEM at 4.5 g/L glucose (HG-DMEM; Invitrogen; Carlsbad, CA, USA). Once the digestion steps were completed, the cell suspension was filtered through a 70 µm nylon strainer, then the cells were counted and seeded at a density of 20,000 cells/cm^2^ and amplified in the presence of high glucose (HG)-DMEM containing 10% FCS (Invitrogen Life Technologies; Carlsbad, CA, USA) and 1% PSA (2D medium). At confluency, the cells were rinsed twice with PBS 1X, then separated by trypsin, counted, and reseeded.

mRNA extracted from eACs at P0 and total protein from equine articular cartilage were used as controls and were prepared from healthy metacarpal joints, as previously described [50,52].

All the methods and procedures were carried out in accordance with relevant guidelines and regulations. The “Comité d’éthique/Agence nationale de sécurité sanitaire/Ecole nationale vétérinaire d’Alfort/Université Paris-est Créteil” (ComEth Anses/ENVA/UPEC) Ethical Committee approved the protocol (Date of approval: 10 March 2015, Permit number: 10/03/15-12).

### 4.2. Co-Culture Experiments

The co-culture experiments were performed in hypoxia (3% O_2_). At P3, eACs were seeded in type I/III collagen sponges (800,000 cells/sponge) or in a monolayer at a density of 20,000 cells/cm^2^ and then placed in 6-well plates. eACs seeded in the biomaterial were cultured in the presence of HG-DMEM, containing 2% FCS, 1% PSA, and 50 µg/mL ascorbic acid (3D medium). Monolayer cells were cultured in the amplification medium (2D medium).

In parallel, at P3, the MSCs were seeded in cell culture inserts (0.4 µm, transparent PET, Falcon, Dominique Dutscher SAS, Brumath, France) at a density allowing to have MSCs/chondrocytes ratios of 1/20, 1/12, 1/8, 1/4. We chose low ratios to correspond more closely to the physiological situation in the cartilage. MSCs were cultured one day in the presence of MSC amplification medium, then the next day, they were transferred to wells containing chondrocytes and their respective media. The media were changed twice a week, and the experiments were stopped on day 7 and day 14.

The D0 corresponds to cells amplified up to P3 as monolayers. We used as positive control chondrocytes cultured in sponges, in the presence of 3D medium supplemented with BMP-2 (50 ng/mL, dibotermine α, Inductos^®^, Medtronic France SAS, Boulogne-Billancourt, France).

### 4.3. Preparation of Conditioned Media

The 2D and 3D media were conditioned by incubation with MSCs cultured in a monolayer at 70/80% of confluency in normoxia (21% O_2_) for 24 h. At the end of the 24 h incubation, the media were recovered, filtered (0.22 µm), and stored at −80 °C. As membrane lysis could occur during repeated freeze–thaw cycles, the harvested medium was divided into several fractions of required volumes in order to be thawed only once just before use.

### 4.4. XTT Assay

The XTT assay (2,3-Bis-2-methoxy-4-nitro-5-sulfophenyl-2H-tetrazolium-5-carboxanilide salt, Sigma-Aldrich; Saint Louis, MO, USA) is a colorimetric test based on the reduction of tetrazolium salts into an orange formazan dye. The reduction occurs only in metabolically active cells. In order to carry out this test, 20,000 eACs/cm^2^ were seeded on a 96-well plate in the presence of HG-DMEM medium supplemented with 10% FCS and PSA. At 80% of confluency, treatments were added and the cells were incubated at 37 °C, 5% CO_2_ for 72 h. Then the cells were incubated in the presence of a mixture containing XTT. Absorbance measurements were made at 450 nm for samples and background noise at 600 nm, the difference between these two measurements determines the metabolic activity. Measurements were made with a microplate reader (Spark control Magellan, TECAN^®^, Lyon, France). All experiments were performed in triplicate and repeated 4 times.

### 4.5. Cytotoxicity Evaluation

The ToxiLight^TM^ assay allows the study of cytotoxicity by quantifying plasma membrane lesions. AK is a protein present in all eukaryotic cells. When the plasma membrane is damaged, AK is rapidly released into the culture medium. The bioluminescence kit is based on the measurement of AK involving two chemical reactions. The first reaction converts adenosine diphosphate (ADP) to adenosine triphosphate (ATP) by the AK released from the damaged cells. The second reaction uses luciferase to catalyze the formation of ATP, it is associated with luminescence emission. The cells are processed under the same conditions as for the XTT test. Eighty microliters of the culture medium from each condition was recovered and transferred to a 96-well plate. One hundred microliters of reagent working solution (AK Reagent Working Solution) (Interchim^®^ Bioluminescence Cytotoxicity Assay Kit, Montluçon, France) were added to each well and incubated for 5 min at room temperature. The bioluminescence is then analyzed by a microplate reader (Spark 10M, TECAN^®^, Lyon, France). All experiments were carried out in triplicate and repeated 4 times.

### 4.6. RNA Isolation and RT-qPCR

Total mRNA was extracted according to the manufacturer’s instructions (RNA-Solv^®^ Reagent, Omega Bio-Tek; Norcross, GA, USA). Then, the reverse transcription was performed from one µg of total RNA using reverse transcriptase (MMLV, Thermo Fisher Scientific; Waltham, MA, USA) and oligo dT (Eurogentec; Liège, Belgium). Reverse transcription-quantitative polymerase chain reactions (RT-qPCRs) were performed using iTaq Universal SYBR Green Supermix, on a CFX96 Touch Real-Time PCR Detection System (Bio-Rad; Hercules, CA, USA). The sequences of the primers are listed in Table 1. We used the 2^−ΔΔCT^ method to calculate the relative gene expression. Each sample was normalized to *β-actin*.

### 4.7. Protein Extraction and Western Blots

At the end of the culture, cells in a monolayer or seeded in 3D scaffolds were washed with phosphate-buffered saline (PBS), and then protein extraction was performed using radioimmunoprecipitation (RIPA) lysis buffer supplemented with several protease inhibitors as previously described [45]. Bradford reagent was used to determine the protein concentration (Bio-Rad; Hercules, CA, USA). The electrophoreses were performed (10 μg of the proteins extracts) using 7.5% polyacrylamide gels (TGX Stain-Free Fast Cast Acrylamide Kit 7.5%; Bio-Rad; Hercules, CA, USA). Then, proteins were transferred to a Polyvinylidenedifluoride membrane (Trans-Blot Turbo RTA Midi PVDF Transfer Kit, Bio-Rad). We used 10% non-fat milk diluted in Tris-buffered saline with 0.1% Tween (TBST, Sigma Aldrich; Saint Louis, MO, USA) to block unspecific binding sites of the membranes. Then, membranes were incubated overnight at 4 °C with primary antibodies and then for 1 h at room temperature with secondary antibodies. All antibodies are listed in Table 2. The chemiluminescence signal (Clarity Western ECL Substrate, Bio-Rad; Hercules, CA, USA) was detected on the ChemiDoc MP Imaging System (Bio-Rad; Hercules, CA, USA).

### 4.8. Scratch Wound Healing Assay

Approximately 22,500 eACs/cm^2^ (P3) were seeded per 96-well plate well (Incucyte Image Lock, Essen Bioscience, Ltd., Royston, UK) in the presence of HG-DMEM medium containing 10% FCS and PSA. Twenty-four hours post-seeding, treatments were added. Thirty hours post-seeding (time = 0 h), the scratch was performed with the Incucyte WoundMaker (Essen Bioscience). Two washing steps with PBS were performed to remove detached cells. Finally, the treatments were added. The relative wound density was assessed, corresponding to the ratio of the area colonized by the cells (time = t) to the total area of the initial scratched region (time = 0 h).

### 4.9. Nanoparticle Tracking Analysis (NTA)

The particles contained in MSC-CM were examined with a NanoSight LM10 (Malvern Instruments Ltd., Malvern, UK). In brief, a monochromatic laser beam at 405 nm was applied to the diluted suspension of MSC-CM and a 60 s video recording of particles moving under Brownian motion was analyzed using NTA software. Each video was analyzed to give the average nanoparticle size with an estimation of the particle concentration in MSC-CM. All measurements were performed in triplicate at a dilution of 1:2.

### 4.10. Transmission Electron Microscopy (TEM)

To monitor the morphology of the particles contained in MSC-CM, TEM was employed using a negative staining method. Briefly, MSC-CM was diluted 200-fold with distilled water. A drop of diluted sample was mixed with a drop of 2% ammonium molybdate used as a negative staining agent for 3 min at room temperature. The mixture was then placed for 5 min on a Formvar-carbon coated copper grid (200 mesh, 3 mm diameter HF 36). The mesh was examined using a CM20 transmission electron microscope (Philips Healthcare; Suresnes, France) operating at 200 kV and micrographs were recorded with an Olympus TEM CCD camera.

### 4.11. Statistical Analyses

All the experiments were repeated at least three times with cells derived from different horses. We used GraphPad Prism 8 (GraphPad Software Inc; San Diego, CA, USA) to perform the Mann–Whitney U-test. A *p*-value of ≤ 0.05 was considered significant.

## Figures and Tables

**Table 1:** Primers used for RT-qPCR.

*ß-Actin.*	Forward	GATGATGATATCGCCGCGCTC
	Reverse	TGCCCCACGTATGAGTCCTT
*Col2a1*	Forward	GGCAATAGCAGGTTCACGTACA
	Reverse	CGATAACAGTCTTGCCCCACTT
*Prg4*	Forward	CTACCACCCAACGCAACAAA
	Reverse	ACTGTTGTCTCCTTATTGGGTGT
*Col1a1*	Forward	TGCCGTGACCTCAAGATGTG
	Reverse	CGTCTCCATGTTGCAGAAGA
*Ki67*	Forward	AAGCTGCACGTTCATGGAGA
	Reverse	ACCCACAGTTCTTCCTCCGA
*Pcna*	Forward	GCGTGAACCTCACCAGTATGT
	Reverse	GCAAATTGCCCAGAAGGCAT
*P53*	Forward	CACCTGAGGTTGGCTCTGAC
	Reverse	GCACAAACACGCACCTCAAA

**Table 2:** Antibodies used for Western blots.

Antibody-Dilution	Supplier
Rabbit anti-bovine type I collagen—1/3000	Novotec; Bron, France
Rabbit anti-human type II collagen—1/1500	
Rabbit anti-human GAPDH—1/5000	Santa Cruz Biotechnology; Dallas, TX, USA
Rabbit anti-human type IIB collagen—1/1500	Covalab; Villeurbanne, France
HRP-conjugated goat anti-rabbit antibody— 1/5000	Jackson Immunoresearch; West Grove, PA, USA

## Data Availability

The data are presented in this study.

## Supplementary Materials

The following supporting information can be downloaded at https://www.mdpi.com/article/10.3390/ijms23105795/s1.
