# Supplementary material for: Bone Marrow MSC Secretome Increases Equine Articular Chondrocyte Collagen Accumulation and Their Migratory Capacities
Source: Int J Mol Sci. 2022 May 21;23(10):5795. doi: 10.3390/ijms23105795 (PMC9146805; doi:10.3390/ijms23105795)
Supplement: Supplementary file 1 [file ijms-23-05795-s001.zip › ijms-1713793-supplementary.pdf]

Bone Marrow MSC Secretome Increases Equine Articular Chondrocyte Collagen Accumulation and Their Migratory Capacities

Romain Contentin [1,†], Manon Jammes [1,†], Bastien Bourdon [1], Frédéric Cassé [1], Arnaud Bianchi [2], Fabrice Audigié [3], Thomas Branly [1], Émilie Velot [2] and Philippe Galéra [1,*]

[1] Normandie Univ, UNICAEN, BIOTARGEN, F-14000 Caen, France; romaincontentin@hotmail.fr (R.C.); manon.jammes@unicaen.fr (M.J.); bastien-bourdon@dielen.fr (B.B.); frederic.casse@unicaen.fr (F.C); tbranly@gmail.com (T.B.); philippe.galera@unicaen.fr (P.G.)

[2] Université de Lorraine, French National Centre for Scientific Research (CNRS), Molecular Engineering and Articular Physiopathology (IMoPA), F-54000 Nancy, France; arnaud.bianchi@univ-lorraine.fr (A.B.); emilie.velot@univ-lorraine.fr (É.V.)

[3] Center of Imaging and Research on Locomotor Affections on Equines (CIRALE), Unit Under Contract 957 Equine Biomechanics and Locomotor Disorders (USC 957 BPLC), French National Research Institute for Agriculture Food and Environment (INRAE), École Nationale Vétérinaire d'Alfort, F-94700 Maisons-Alfort, France; fabrice.audigie@vet-alfort.fr (F.A)

* Correspondence: philippe.galera@unicaen.fr

† These authors contributed equally to the work.

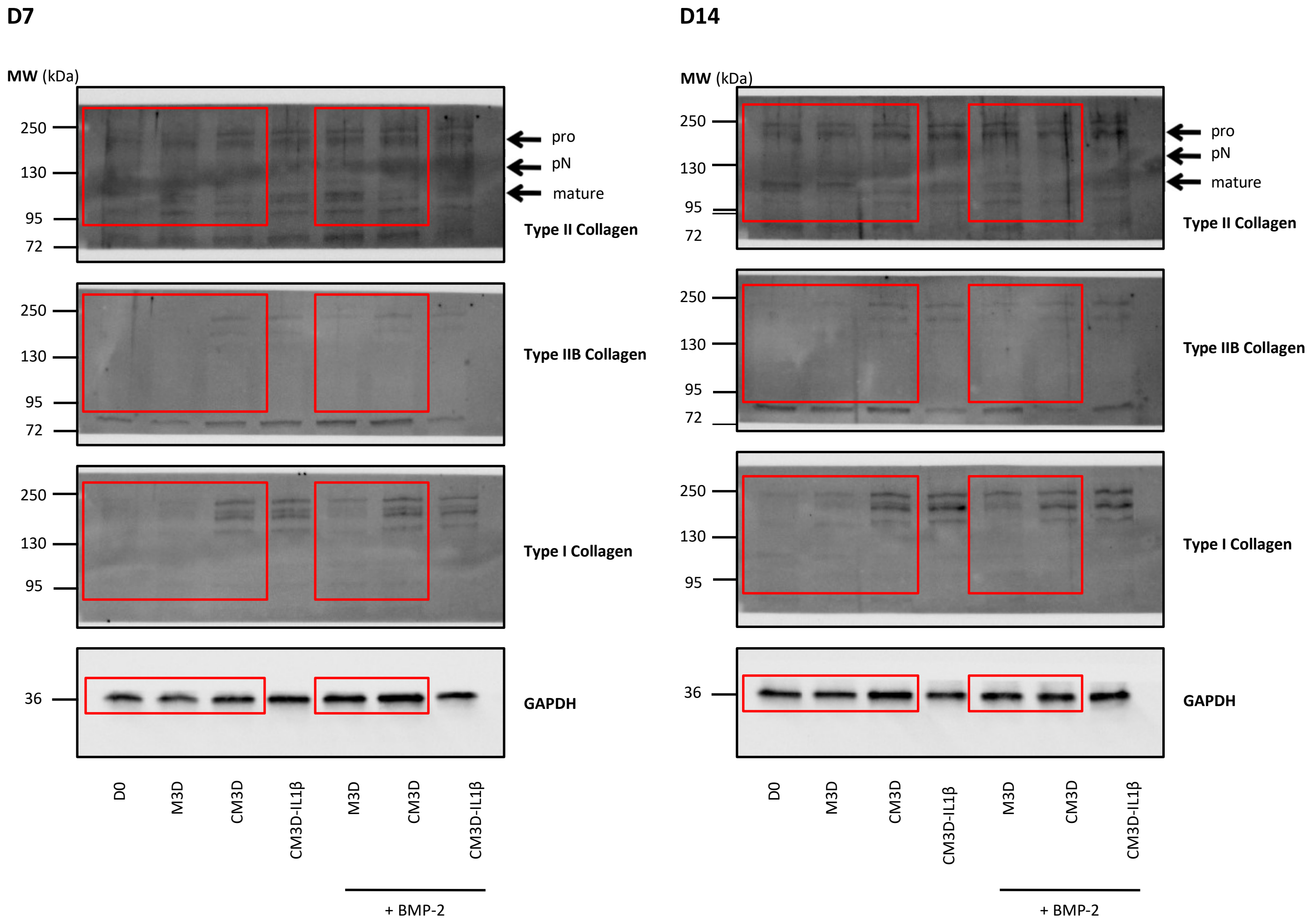

Figure S1. Complete gel and PVDF membranes analyzed in western-blot presented in Figure 4B. Images were captured with Chemidoc MP Imaging-System (Bio-Rad). Molecular weights (kDa) are indicated on the left of all blots. The cropped images shown in Figure 4B are highlighted in red boxes.

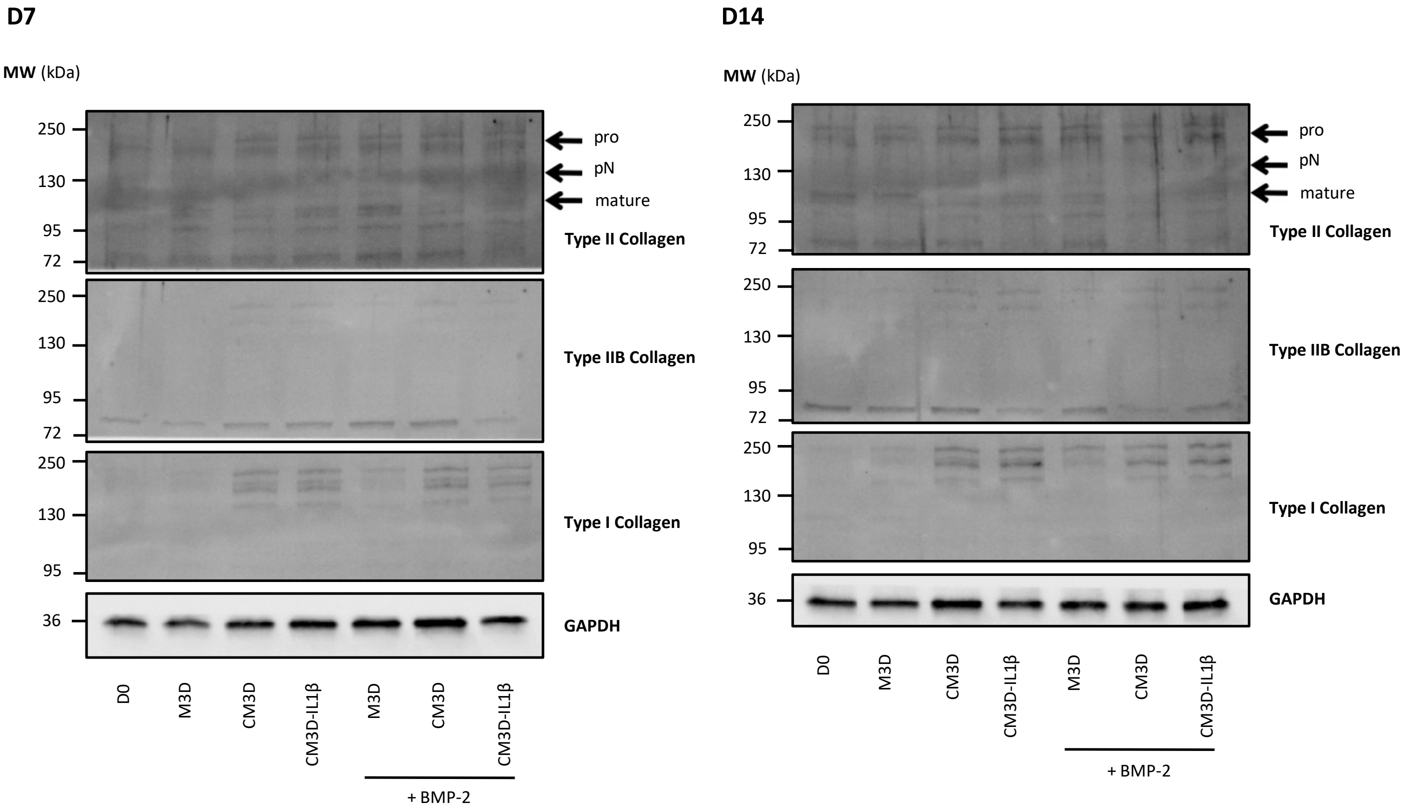

Figure S2. Under-exposure of the blots presented in Figure 4B.
Images were captured with Chemidoc MP Imaging-System (Bio-Rad). Molecular weights (kDa) are indicated on the left of all blots.

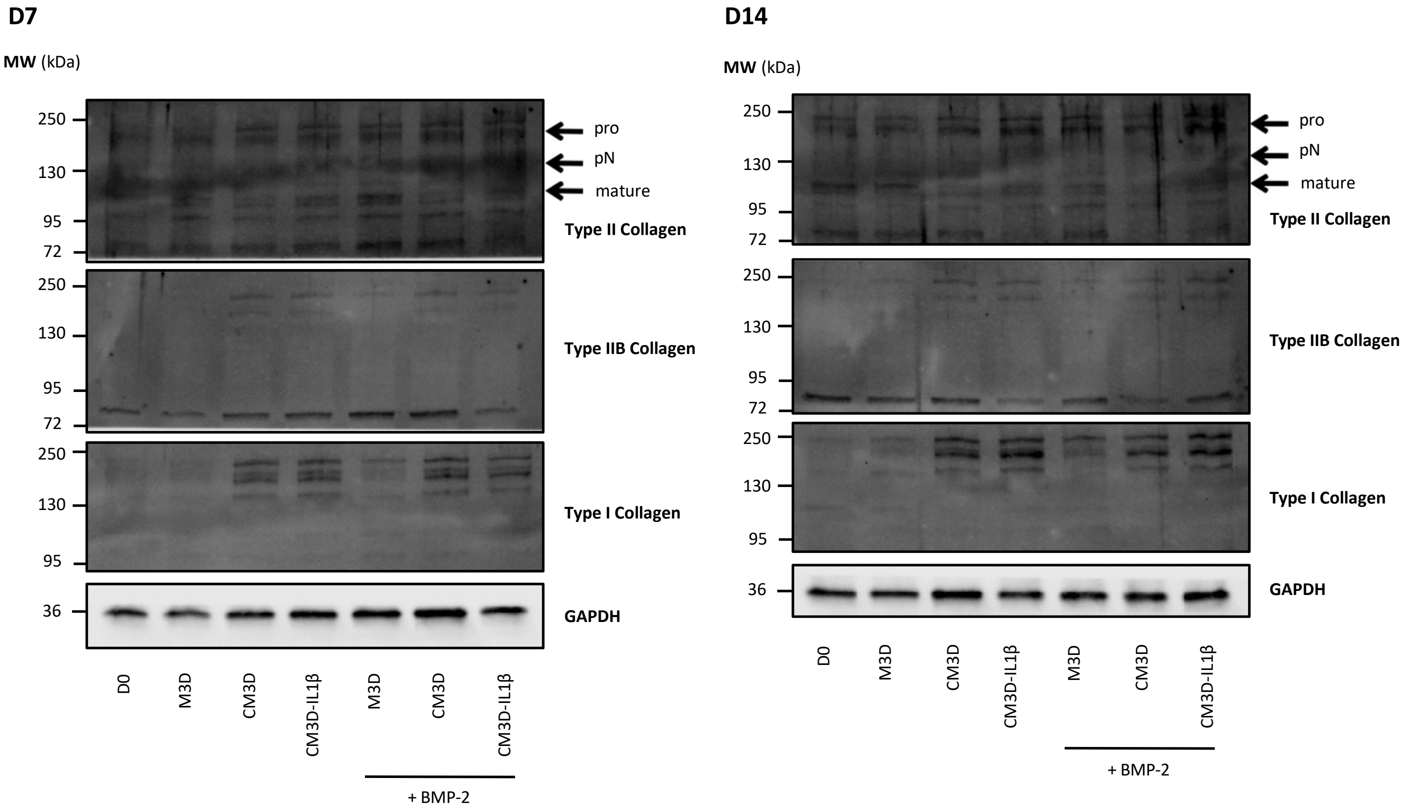

Figure S3. Over-exposure of the blots presented in Figure 4B.
Images were captured with Chemidoc MP Imaging-System (Bio-Rad). Molecular weights (kDa) are indicated on the left of all blots.

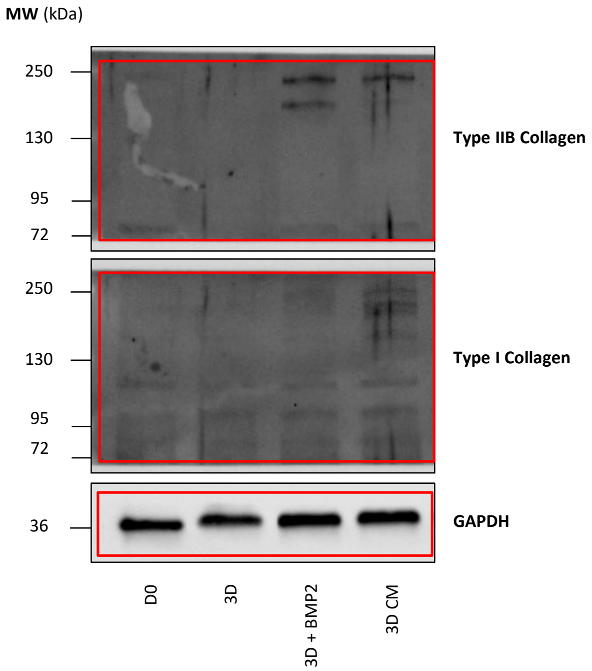

Figure S4. Complete gel and PVDF membranes analyzed in western-blot presented in Figure 4C. Images were captured with Chemidoc MP Imaging-System (Bio-Rad). Molecular weights (kDa) are indicated on the left of all blots. The cropped images shown in Figure 4C are highlighted in red boxes.

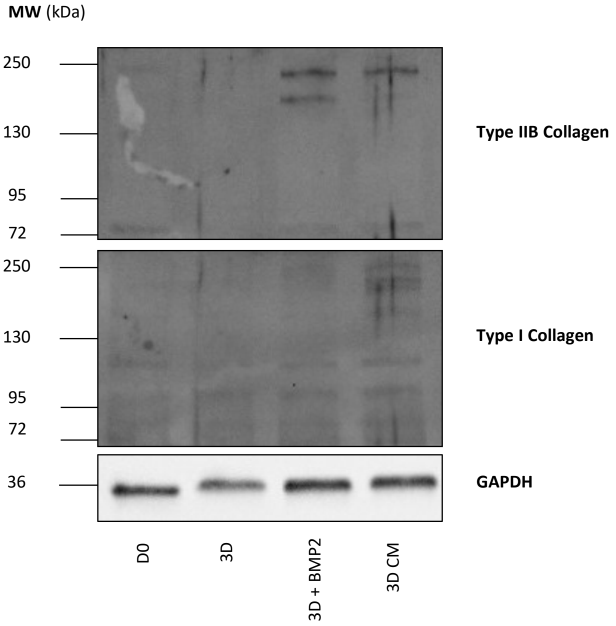

Figure S5. Under-exposure of the blots presented in Figure 4C.
Images were captured with Chemidoc MP Imaging-System (Bio-Rad). Molecular weights (kDa) are indicated on the left of all blots.

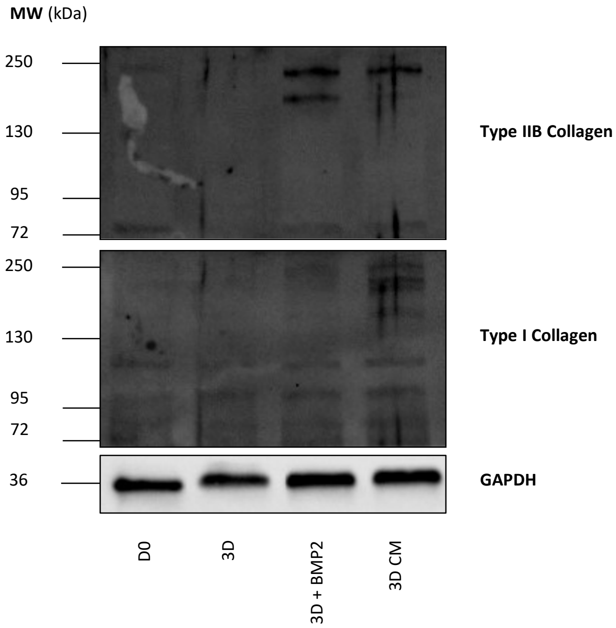

Figure S6. Over-exposure of the blots presented in Figure 4C.
Images were captured with Chemidoc MP Imaging-System (Bio-Rad). Molecular weights (kDa) are indicated on the left of all blots.
